# Evaluation of bone depth, cortical bone, and mucosa thickness of palatal posterior supra-alveolar insertion site for miniscrew placement

**DOI:** 10.1186/s40510-022-00412-9

**Published:** 2022-06-06

**Authors:** Riccardo Nucera, Elia Ciancio, Giuliano Maino, Serena Barbera, Emanuela Imbesi, Angela Mirea Bellocchio

**Affiliations:** 1 Department of Biomedical and Dental Sciences and Morphofunctional Imaging, Section of Orthodontics, School of Dentistry, University of Messina, Messina, Italy; 2 Postgraduate School of Orthodontics, University of Ferrara, Ferrara, Italy

**Keywords:** Orthodontic miniscrew, Palatal expansion, CBCT, Palatal miniscrew insertion site, TADs, Palatal mucosa, Cortical bone thickness, Palatal bone depth

## Abstract

**Background:**

The use of palatal miniscrew offers the possibility to improve the effectiveness of orthodontic expansion devices. Palatal expanders supported by miniscrew can be applied with different clinical protocols. Some authors proposed the use of four palatal miniscrews during miniscrew-supported palatal expansion to maximize skeletal effects in young adults’ treatment. However, bone availability decreases in the posterior paramedian palatal regions, making the positioning of the two-posterior paramedian palatal miniscrews challenging, when it is performed avoiding nasal cavities invasion. Some authors proposed miniscrews insertion in a specific region located laterally to the palatal process of the maxillary bone, and apically relatively to the dento-alveolar process. The aim of this study was to evaluate the bone thickness, cortical bone thickness, and mucosae depth of this anatomical site that, in this study, was defined as palatal posterior supra-alveolar insertion site.

**Results:**

The evaluation of bone availability of palatal posterior supra-alveolar insertion site at different antero-posterior levels showed that the maximum amount of total bone thickness was found between the second premolar and the first molar. At this level total bone, thickness is significantly (*p* < .05) greater compared to the other sagittal sites and it offers on average around 2 mm of extra bone depth for miniscrew placement. Cortical bone thickness is adequate for primary miniscrew stability. Overall, cortical bone thickness considered at different insertion sites showed significant statistically (*p* < .05) differences. The findings of this study showed that palatal mucosa is particularly thick with average values ranging from 4 to 7 mm, and its extension ultimately affects miniscrew length selection. Palatal mucosa thickness showed no clinically significant differences comparing different sagittal and vertical insertion sites. Data also showed that palatal mucosal thickness slightly significantly increases (*p* < .05) with the inclination of the insertion axis relative to the occlusal plane. Finally, study findings showed that vertical growth pattern can significantly affect considered outcomes (*p* < .05).

**Conclusions:**

Palatal posterior supra-alveolar insertion site is an appropriate site for posterior insertion of palatal miniscrews. Considering high anatomical variation preliminary CBCT evaluation is important to achieve optimal miniscrew placement.

**Supplementary Information:**

The online version contains supplementary material available at 10.1186/s40510-022-00412-9.

## Background

Since their introduction in clinical practice, orthodontic miniscrew, also called temporary anchorage devices (TADs), expanded the orthopedic possibilities of orthodontic appliances [1, 2].

The palatal vault is a suitable site for the miniscrews insertion [3–5]. The use of palatal miniscrew offers the possibility to improve the effectiveness of expansion devices [2, 6–9].

Palatal expansion devices supported by TADs can be applied with different protocols. The most common miniscrews configuration supporting maxillary expansion are: two palatal miniscrews on the anterior side of the palate [10, 11] or four miniscrews, two on the anterior side, and two more posteriorly in the palatal vault [12, 13].

Some authors suggest the use of four palatal miniscrews during miniscrew-supported palatal expansion to maximize skeletal effects in the treatment of young adults [2, 14, 15].

However, the vertical bone thicknesses of the palate vault decrease in the posterior paramedian palatal zones at the molar sagittal level [3–5]. This aspect makes the positioning of the two posterior paramedian palatal miniscrews challenging when it is performed avoiding nasal cavities invasion.

To overcome these issues some authors proposed miniscrews insertion in the palatal posterior vault positing a miniscrew body in a specific region located laterally to the palatal process of the maxillary bone, and apically relatively to the dento-alveolar process above the roots of the first maxillary molars. This specific insertion site has been used by some authors [13, 16–19], but it has not been given a specific name.

The aim of this study was to evaluate the bone thickness, cortical bone thickness, and mucosae depth of this site that, in this study, it was defined as Palatal Posterior Supra-Alveolar Insertion Site (PPSAIS). To the best of our knowledge, there are no studies that specifically investigate the anatomical characteristics of the PPSAIS.

## Methods

This retrospective cross-sectional CBCT study was reviewed and approved by the Ethics Committee of the University of Messina (prot. no 33-2020). It was conducted according to the principles of the Declaration of Helsinki (1964) and its later amendments.

The archives of three private practices and one university clinic were searched, and the pre-treatment records of patients showing the following inclusion criteria were selected: Caucasian subjects with intact permanent dentition (excluding third molars); constricted maxilla with a distance between the closest points of the upper first molar crowns (i.e., transpalatal width) less than 34 mm and mono or bilateral posterior crossbite malocclusion; subjects that underwent CBCT examination for palatal miniscrew placement planning or surgical assisted palatal expansion treatment planning; and subjects aged between 14 and 25 y.o.

Applied exclusion criteria were: verified diagnosis of craniofacial syndromes such as cleft palate, cysts, or maxillary tumors; confirmed diagnosis of periodontal disease; and history of previous orthodontic treatment.

Fifty-five patients (30 females, 25 males) fulfilled the inclusion criteria, and their CBCT files, their lateral cephalometric radiographs, and maxillary digital models were checked for quality and integrity. Patients were divided by gender into two numbered groups, and each group was sorted by age. A random sequence generator was used to create two lists of randomized numbers sequence of 30 and 25 numbers.

To set the sample size, a preliminary power analysis calculation was performed, based on 10 first evaluated cases. Total bone depth outcome, measured with 45° inclination compared to the occlusal plane, was used for power analysis calculation.

Specifically, the considered data were: 8.11 mm (bone depth between second premolar and first molar), 6.23 mm (bone depth between first molar and second molar), 3.15 (common standard deviation). The power level has been set to 80% and the significance level to 0.5. The results of the power analysis showed that a sample of 45 cases was required to achieve adequate study power.

The first 23 and 22 numbers of both previously mentioned groups were selected, and the relative patient's records were included. By this procedure, case enrollment was performed according to a balanced randomization system according to patient gender.

The final sample included 45 subjects (mean age 18.9 ± 3.1 y.o), 22 male subjects (mean age 18 ± 2.7 y.o), and 23 female subjects (mean age 19.8 ± 3.2 y.o). The sample included seventeen subjects affected by skeletal Class I, seventeen subjects exhibiting a skeletal class II and eleven subjects showing a class III skeletal relationship. Regarding the vertical skeletal pattern, twenty subjects exhibited a mesodivergent pattern, fifteen patients were hyperdivergent and ten subjects showed a hypodivergent skeletal pattern. No subject showed signs of obesity such as the double chin or soft tissue perio-oral abundance.

**Fig. 1:**
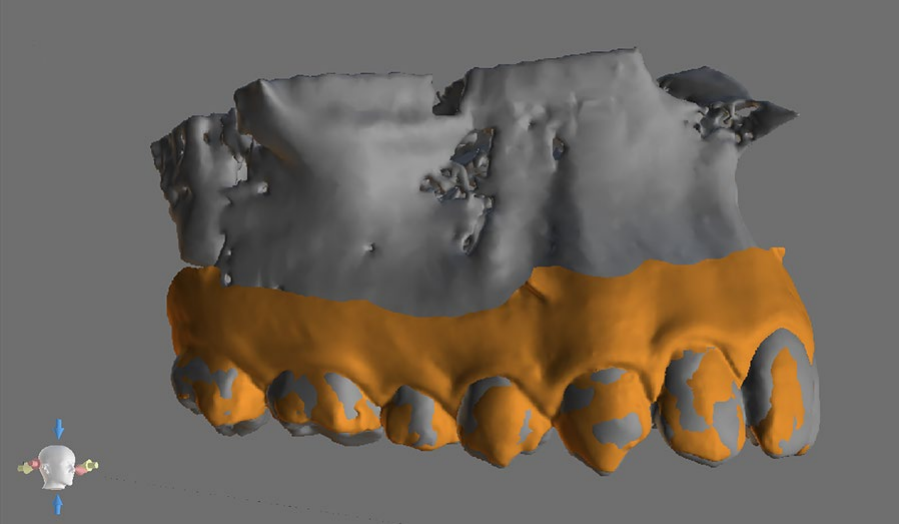
CBCT scan and maxillary digital model superimposition

CBCT and lateral cephalograms records were generated by Kavo OP 3D Pro CBCT scanner (Kavo Dental Technologies, Imaging Sciences International, Hatfield, PA, USA). CBCT examinations were performed setting the following parameters acquisition: 90 kv, 6.3–8 mA, 5–8 s exposure time. CBCT examinations were exported as DICOM file format and then imported in BlueSkyPlan software (BlueSkyBio LLC, V4.7–64 bit, Libertyville, IL, USA). The import of CBCT examinations was performed by reorienting the occlusal plane with a parallel disposition compared to the axial plane. Lateral cephalograms were executed with the following parameters: 90kv, 10 mA, 16 s. Cephalometric tracing, landmark identification, and measurements of the following cephalometric outcomes were performed using a dedicated software (WebCeph, AssembleCircle Corp., Seongnam, Republic of Korea): ANB(°), SN-GoGn(°). Digital maxillary models were obtained by intro-oral scanner acquisition (Medit i500, Medit, Seoul, Republic of Korea) and exported in STL file format. Subsequently, maxillary digital models were imported in BlueSkyPlan software and superimposed to CBCT volumes (Fig. 1). The software (internationally registered as a medical device) used an algorithm to automatically superimpose the digital maxillary model and the CBCT volume scan. At the end of the superimposition process (Fig. 1), the accuracy of the superimposition was checked, by visual examination on conventional 3 view planes (coronal, sagittal, and axial).

Outcome measurements were performed only on one side of the evaluated cases. The evaluated side (right or left) was selected by a randomized procedure. Forty-five included cases were sorted by age and numbered in sequence. A balanced random sequence with 45 values of “0” and “1” was generated. The created random sequence was associated with the list of included subjects. Cases associated with value “0” were right-sided evaluated, and subjects associated with value “1” were left-sided evaluated.

All outcome evaluations were performed in three different coronal scans located at different antero-posterior levels: interproximal contact point between the second premolar and the first molar (P2-M1), at the upper first molar furcation (M1F), and interproximal contact between the first and the second molar (M1-M2).

**Fig. 2:**
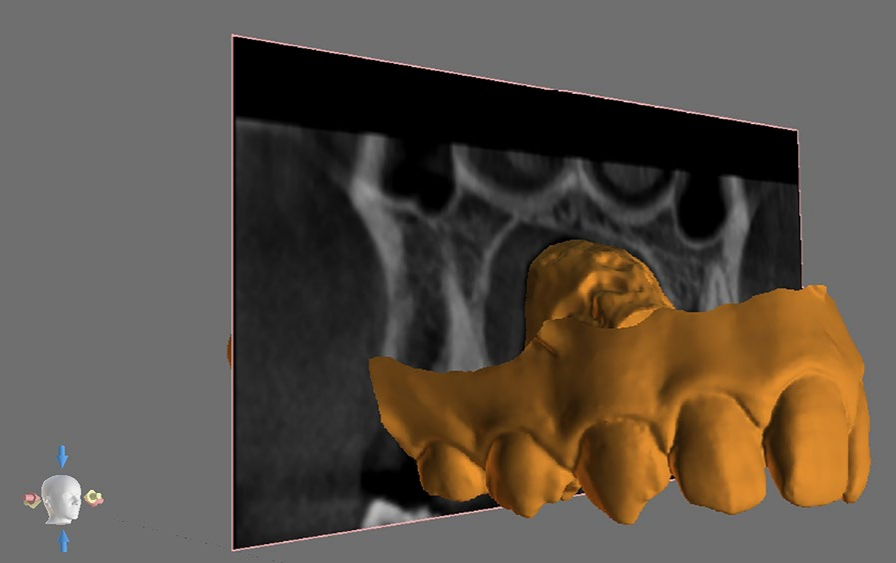
Outcome evaluation was performed in coronal scans set at different antero-posterior level: interproximal contact point between the second premolar and the first molar (P2-M1), at the upper first molar furcation (M1F) and interproximal contact between the first and the second molar (M1-M2)

The coronal plane selection was performed using the software interface with the coronal scan view and the superimposed 3D view showing the maxillary digital stereolithographic model (Fig. 2).

**Fig. 3:**
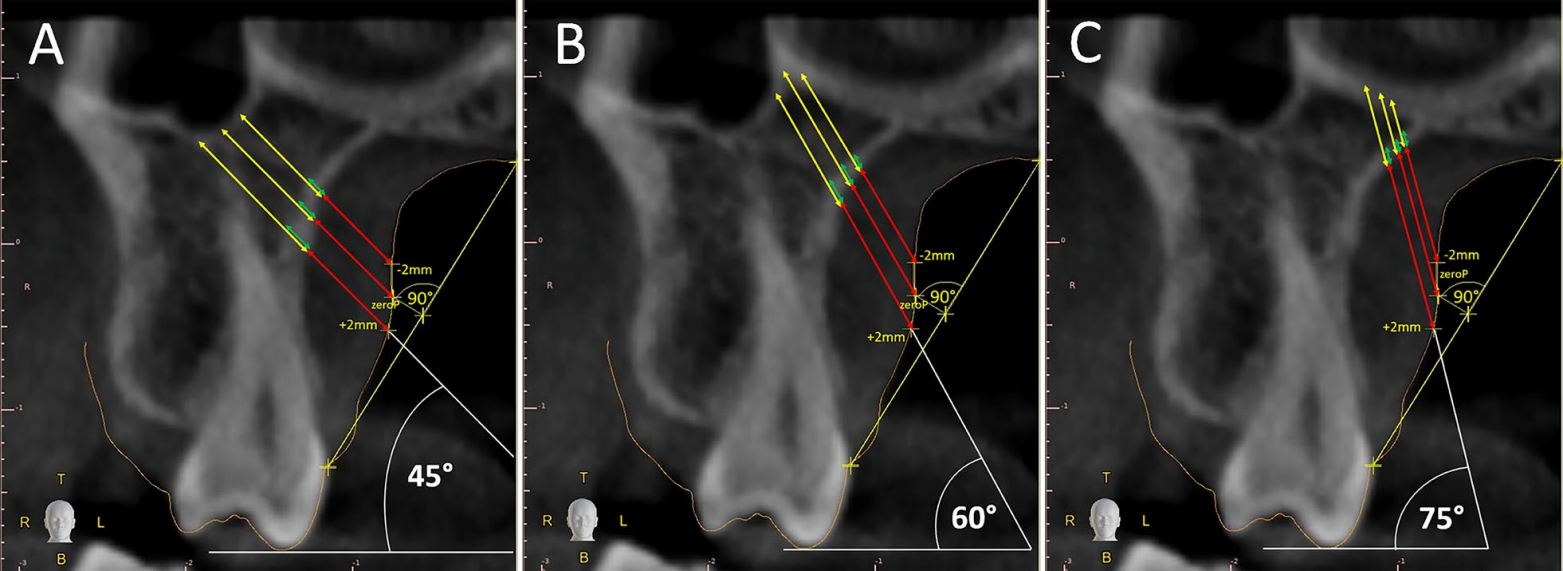
The following outcomes were evaluated: total bone depth (yellow segment), cortical bone thickness (green segment), and palatal mucosa thickness (red segment). Outcomes measurement was performed on three straight lines traced passing through the three landmarks identified on the palatal mucosa profile (− 2P, zeroP, and + 2P). Three sets of lines with different angulation to the occlusal plane were considered: 45° (**a**), 60° (**b**), and 75° (**c**). This evaluation procedure was repeated for the three considered coronal scans. Overall, for each patient 27 insertion sites were evaluated, and 81 outcomes were measured

Each selected coronal scan (Fig. 3) was used to identify three reference landmarks along the palatal mucosa profile vault (orange line). To identify the first landmark, a segment was traced connecting the free gingival margin with mucosa overlying the mid-palatal suture (Fig. 3). This segment was defined palatal slope segment (PSS). The middle point of PSS was identified, and its 90° projection was traced to the palatal mucosa profile. The point located at the intersection of the 90° middle point projection and the palatal mucosa profile was the first identified landmark and it was named “zeroP” (Fig. 3a).

The second landmark was identified 2 mm apically to zeroP along the palatal mucosa profile and was named “− 2P,” and the third landmark was identified 2 mm coronally to zeroP along the palatal mucosa and was named “+ 2P.”

To perform outcome measurement, three straight lines were traced passing through the three identified landmarks (− 2P, zeroP, and + 2P) and with a 45° inclination compared to the occlusal plane (Fig. 3a).

Along these three straight lines, the following outcomes were measured: total bone depth (yellow segment), cortical bone thickness (green segment), and palatal mucosa thickness (red segment).

The above-mentioned outcomes were also measured on six other straight lines passing through the three identified landmarks (+ 2P, zeroP, and − 2P) and with a 60° and 75° inclination compared to the occlusal plane (Fig. 3b, c).

Consequently, outcomes were measured with 3 different angulations (45°, 60°, and 75°) and at different vertical levels (− 2P, zeroP, and + 2P) in three different antero-posterior sites (P2-M1, M1F, and M1-M2). According to this methodological approach, 27 insertion sites were evaluated for each patient.

For every insertion site, the following outcomes were measured: total bone depth (TBD), cortical bone thickness (CBT), and mucosa thickness (MT) for total of 81 measured outcomes for each included subject.

Descriptive statistics and inferential statistics were performed using SPSS statistics software (version 25.0; IBM Corporation, Armonk, NY). The significance levels were set at *p* < 0.05. Preliminary data analysis included normal distribution (Shapiro–Wilk test) and equality of variances (Levene’s test) evaluation. Data showed a non-normal distribution for the evaluated outcomes. Consequently, nonparametric Kruskal–Wallis multiple comparison test and Dunn–Bonferroni post hoc tests were used for inferential statistics evaluation. Spearman correlation coefficient was used to investigate the association between sample age and total bone depth. Descriptive statistics were performed calculating mean, standard deviation, maximum, and minimum values for each considered outcome.

To assess the methodological error, the digital model and CBCT superimposition, scan view identification, and outcome measurements were repeated one week apart for 10 patients randomly selected. Paired t tests and intraclass correlation coefficient (ICCs) were used to assess the intraoperator reliability. The magnitude of the random error was assessed using the Dahlberg formula. No significant differences (*p* < 0.05) were noticed between the two readings; all measurements were reliable, with the ICC varying from 0.78 to 0.85. Random error ranged from 0.15 to 0.34 mm.

## Results

Descriptive and inferential statistics of data for all considered outcomes are reported in Table 1. Descriptive statistics of every outcome independently considered reporting: mean, standard deviation, minimum, and maximum values, are reported in Tables 2, 3, and 4 for mucosa thickness, cortical bone thickness, and total bone depth, respectively. Kruskal–Wallis multiple comparison tests evaluating the impact of the sagittal skeletal relationship on considered outcomes showed no significant result. No correlation between sample age and total bone thickness was found. The remaining inferential statistics analysis conducted independently for every considered outcomes is reported in Additional file 1: Table S1 (mucosa thickness), Additional file 1: Table S2 (cortical bone thickness), and Additional file 1: Table S3 (total bone depth).

**Table 1:** Descriptive and inferential statistics with pooled groups of total bone depth (TBD), cortical bone thickness (CBT) and mucosa thickness (MT) outcomes

	TBD		CBT		MT	
	Mean (SD)	Min–Max	Mean (SD)	Min–Max	Mean (SD)	Min–Max
zeroP	5.77(3.52)	0.78–26.32	1.47(0.65)	0.47–6.16	5.63(1.61)	0.83–12.11
+ 2 mm	6.43(3.51)	1.04–17.66	1.67(0.70)	0.55–5.10	5.26(1.70)	1.66–11.59
Multiple comparison test*	*p* < .001		*p* < .001		*p* = .001	
−2 mm Vs zeroP**	*p* < .05		*p* < .001		NS	
zeroP Vs + 2 mm**	*p* < .001		*p* < .001		*p* < .001	
2 mm Vs −2 mm**	*p* < .05		*p* < .001		NS	

	Mean (SD)	Min–Max	Mean (SD)	Min–Max	Mean (SD)	Min–Max
45°	6.11(3.54)	0.92–26.32	1.55(0.62)	0.43–4.68	4.83(1.40)	1.62–14.05
60°	5.86(3.58)	0.78–19.57	1.43(0.59)	0.43–4.33	5.42(1.47)	1.78–10.67
75°	5.41(3.24)	1–17.57	1.41(0.69)	0.47–6.16	6.07(1.74)	0.83–12.11
Multiple comparison test*	*p* < .05		*p* < .001		*p* < .001	
75° vs 60°**	NS		NS		*p* < .001	
75° vs 45°**	*p* < .05		*p* < .001		*p* < .001	
60° vs 45°**	NS		*p* < .05		*p* < .001	

	Mean (SD)	Min–Max	Mean (SD)	Min–Max	Mean (SD)	Min–Max
P2-M1	7.2(4.13)	1–26.32	1.54(0.57)	0.53–4.68	5.37(1.37)	2.52–12.11
M1F	5.04(2.85)	0.92–14.85	1.38(0.57)	0.43–4.64	5.33(1.50)	2.19–10.65
M1-M2	5.12(2.809	0.78–15.87	1.47(0.75)	0.43–6.16	5.62(1.93)	0.83–14.05
Multiple comparison test*	*p* < .001		*p* < .001		NS	
P2-M1 vs M1-M2**	*p* < .001		*p* = .001		NS	
P2-M1 vs M1F**	*p* < .001		*p* < .001		NS	
M1-M2 vs M1F**	NS		NS		NS	

	Mean (SD)	Min–Max	Mean (SD)	Min–Max	Mean (SD)	Min–Max
Hypodivergent	6.62(3.7)	1.01–26.32	1.62(0.6)	0.58–4.68	4.90(1.1)	2.52–8.79
Mesodivergent	5.50(3.4)	0.92–19.57	1.40(0.6)	0.43–4.64	5.56(1.6)	2.19–12.11
Hyperdivergent	5.64(3.2)	0.78–17.48	1.47(0.8)	0.43–6.16	5.67(1.9)	0.83–14.5
Multiple comparison test*	*p* < .001		*p* < .001		*p* < .001	
Hypodivergent Vs Mesodivergent**	*p* < .001		*p* < .001		*p* < .001	
Mesodivergent Vs Hyperdivergent**	NS		NS		NS	
Hypodivergent Vs Hyperdivergent**	*p* < .05		*p* < .001		*p* < .001	

Values are reported in millimeters (mm). *Kruskal–Wallis multiple comparison test and **Dunn–Bonferroni post hoc tests were used for inferential statistics evaluating the following independent variables: corono-apical evaluation level, miniscrew axis of inclination, inter-radicular location, and mandibular divergency

**Table 2:** Descriptive statistics of total bone depth outcomes

	Total bone depth											
	Mean			Standard deviation			Minimum			Maximum		
	P2-M1	M1F	M1-M2	P2-M1	M1F	M1-M2	P2-M1	M1F	M1-M2	P2-M1	M1F	M1-M2
− 2P(45°)	7.1	4.9	5.0	4.0	2.8	3.0	1.4	0.9	1.0	15.5	11.1	11.6
zeroP(45°)	7.6	4.9	5.6	4.6	2.7	2.5	1.6	1.1	1.0	26.3	12.8	12.0
+ 2P(45°)	8.0	5.8	6.1	4.2	3.0	3.2	1.6	1.1	1.1	17.7	12.2	13.6
− 2P(60°)	6.9	5.1	5.1	4.3	3.3	2.9	1.1	1.0	1.2	19.6	13.8	14.3
zeroP(60°)	7.5	5.0	4.8	4.3	3.1	2.9	1.3	1.0	0.8	19.0	14.9	11.6
+ 2P(60°)	8.0	5.1	5.4	4.1	2.6	2.7	1.4	1.1	1.0	17.2	12.1	11.5
− 2P(75°)	5.0	4.1	3.6	2.8	2.2	1.8	1.0	1.2	1.2	11.9	8.7	10.0
zeroP(75°)	6.8	4.8	5.0	4.0	2.7	2.9	1.2	1.1	1.0	17.6	10.7	15.9
+ 2P(75°)	8.3	5.7	5.5	4.0	3.0	2.8	1.6	1.0	1.4	17.2	12.4	11.2

Values are reported in millimeters (mm). Outcomes were evaluated between second premolar and the first molar (P2-M1), at the furcation of the first molar (M1F) and between the first molar and the second molar (M1-M2). Insertion axes were traced passing through 3 landmarks: zero point (zeroP), 2 mm cranial to zeroP (− 2P), and 2 mm caudal to zeroP (+ 2P). Insertion axes were also traced at three different angulations (45°, 60°, and 75°) compared to the occlusal plane

**Table 3:** Descriptive statistics of cortical bone thickness outcomes

	Cortical bone thickness											
	Mean			Standard deviation			Minimum			Maximum		
	P2-M1	M1F	M1-M2	P2-M1	M1F	M1-M2	P2-M1	M1F	M1-M2	P2-M1	M1F	M1-M2
− 2P(45°)	1.5	1.3	1.3	0.4	0.5	0.7	0.7	0.4	0.4	2.6	2.4	3.9
zeroP(45°)	1.7	1.5	1.7	0.5	0.6	0.8	0.6	0.7	0.5	3.3	3.1	4.6
+ 2P(45°)	1.8	1.7	1.6	0.6	0.7	0.6	0.8	0.7	0.7	4.7	4.4	3.0
− 2P(60°)	1.3	1.2	1.2	0.5	0.5	0.4	0.6	0.4	0.5	2.9	2.4	2.3
zeroP(60°)	1.6	1.3	1.4	0.6	0.5	0.7	0.6	0.6	0.6	3.2	2.6	4.3
+ 2P(60°)	1.6	1.5	1.8	0.6	0.5	0.8	0.7	0.6	0.8	3.6	2.5	3.9
− 2P(75°)	1.2	1.2	1.2	0.4	0.5	0.4	0.5	0.5	0.6	2.2	2.3	2.4
zeroP(75°)	1.4	1.3	1.5	0.4	0.5	1.0	0.7	0.5	0.5	2.4	3.0	6.2
+ 2P(75°)	1.9	1.6	1.6	0.6	0.7	1.0	0.7	0.6	0.6	3.4	4.6	5.1

Values are reported in millimeters (mm). Outcomes were evaluated between second premolar and first molar (P2-M1), at the furcation of the first molar (M1F) and between the first molar and the second molar (M1-M2). Insertion axes were traced passing through 3 landmarks: zero point (zeroP), 2 mm cranial to zeroP (− 2P), and 2 mm caudal to zeroP (+ 2P). Insertion axes were also traced at three different angulations (45°, 60°, and 75°) compared to the occlusal plane

**Table 4:** Descriptive statistics of mucosa thickness outcomes

	Mucosa thickness											
	Mean			Standard deviation			Minimum			Maximum		
	P2-M1	M1F	M1-M2	P2-M1	M1F	M1-M2	P2-M1	M1F	M1-M2	P2-M1	M1F	M1-M2
− 2P(45°)	4.9	5.1	6.2	1.0	1.4	2.0	3.6	2.8	1.6	8.1	8.7	14.1
zeroP(45°)	4.7	4.9	5.1	0.9	1.1	1.3	2.9	3.1	1.8	6.6	7.6	8.8
+ 2P(45°)	4.5	4.1	4.0	1.0	0.9	1.4	2.5	2.2	1.7	7.4	6.7	9.2
− 2P(60°)	5.4	5.3	5.7	1.1	1.4	1.9	3.4	2.4	2.1	7.8	8.3	9.9
zeroP(60°)	5.3	5.7	6.1	1.2	1.5	1.9	3.7	3.2	1.8	8.4	10.7	10.4
+ 2P(60°)	5.2	5.1	5.1	1.2	1.2	1.5	3.3	2.9	2.2	8.8	7.9	8.4
− 2P(75°)	5.7	5.2	5.3	1.3	1.5	1.7	3.3	2.3	1.6	7.8	9.1	8.7
zeroP(75°)	6.5	6.2	6.3	1.6	1.5	2.1	3.7	3.1	0.8	12.1	9.7	12.0
+ 2P(75°)	6.2	6.4	6.9	1.6	1.5	2.1	3.6	3.2	2.8	10.1	10.2	11.6

Values are reported in millimeters (mm). Outcomes were evaluated between second premolar and the first molar (P2-M1), at the furcation of the first molar (M1F) and between the first molar and the second molar (M1-M2). Insertion axes were traced passing through 3 landmarks: zero point (zeroP), 2 mm cranial to zeroP (− 2P), and 2 mm caudal to zeroP (+ 2P). Insertion axes were also traced at three different angulations (45°, 60°, and 75°) compared to the occlusal plane

## Discussion

To the best of our knowledge, this is the first study that specifically evaluates the anatomical characteristics of the Palatal Posterior Supra-Alveolar Insertion Site (PPSAIS).

PPSAIS is located cranially compared to the alveolar bone of the maxillary posterior dentition and laterally to the palatal process of the maxillary bone. It is demarcated latero-cranially by maxillary sinus cortical bone, medio-cranially by the cortical bone of the nasal cavity, medially by the palatal cortical bone, and caudally by the alveolar process (Fig. 3).

Consequently, PPSAIS overall presents three cortical plates (sinus, nasal, and palatal) and well-represented trabecular bone within them. The presence of several cortical plates potentially offers the possibility to have a better miniscrew stabilization.

It can be considered a strategic insertion site for palatal posterior miniscrew application. Some authors used this insertion site to obtain several orthopedic and orthodontic treatment effects such as skeletal palatal expansion [13, 15, 20], intrusion of maxillary posterior dental elements [18, 19, 21, 22], and upper molar distalization [23].

PPSAIS offers numerous potential benefits. It is accessible on the palatal side of maxillary arch, and consequently, it presents attached gingiva. This characteristic seems to offer an advantage in terms of miniscrew survival [24].

Moreover, it is located in the posterior part of the palate, and this aspect makes it suitable for the application of more effective posterior expansion forces [13, 15, 20], distalization forces [23], and intrusion forces for posterior maxillary dentition [18, 19, 21, 22].

Literature showed that different authors published case reports using PPSAIS at different antero-posterior levels. Specifically, some authors placed TADs between the second premolar and the first molar [13, 15]; others opted for placing TADs between the second molar and the first molar [18, 19].

To analyze the ideal characteristics of PPSAIS, it is essential to evaluate what are the insertion sites that offer an adequate amount of total bone thickness for optimal miniscrew primary stability.

In this study, the evaluation of bone availability at different antero-posterior levels showed that the maximum amount of total bone thickness was found between the second premolar and the first molar (P2-M1). At this level, total bone thickness is statistically significantly greater compared to the other sagittal sites (Table 1 and Additional file 1: Table S1) and on average around 2 mm ticker (Tables 1 and 2). This datum indicated a general bone reduction in the posterior part of the palate.

No significant differences were noticed comparing the total bone thickness at the first molar furcation site (M1F) and to the M1-M2 insertion site (Additional file 1: Table S1). This finding could be related to the presence of the upper first molar palatal root that could reduce the overall quantity of bone at furcation (M1F).

To support clinicians during miniscrew insertion, this study was conducted describing characteristics of PPSAIS using anatomical references visible during miniscrew insertion procedures.

For this purpose, in each considered cross-sectional scan, it was used as starting insertion point the 90° projection on the palatal mucosa of the middle point of the segment connecting the free gingival margin with mucosa overlying mid-palatal suture (Fig. 3). This point, named “zeroP,” can be approximately visualized by the clinician during miniscrew insertion, it is located in the transition zone of the palate and dento-alveolar process, and it was used as reference point to perform the outcomes evaluation at different corono-apical levels.

In fact, outcomes were also evaluated 2 mm cranial to this starting point along the profile of palatal mucosa and 2 mm caudal to it. Statistically, the maximum average amount of bone was found 2 mm caudal (+ 2P) to zeroP (8.3 mm) at P2M1 and with 75° of insertion axes (Table 2). Two millimeters cranial to zeroP (− 2P), the amount of bone seems to be significantly reduced (Table 1 and Additional file 1: Table S1).

The amount of total bone could be affected by the insertion inclination axis. However, comparing the 3 axes inclination (45°, 60°, and 75°), no significant differences were found. All the evaluated sites showed on average 8 mm of total bone thickness. Moreover, considering descriptive statistics data evaluation, it is possible to conclude that the amount of total bone varies according to individual patient characteristics. Consequently, the ideal miniscrew inclination could be obtained only with a preliminary CBCT evaluation.

**Fig. 4:**
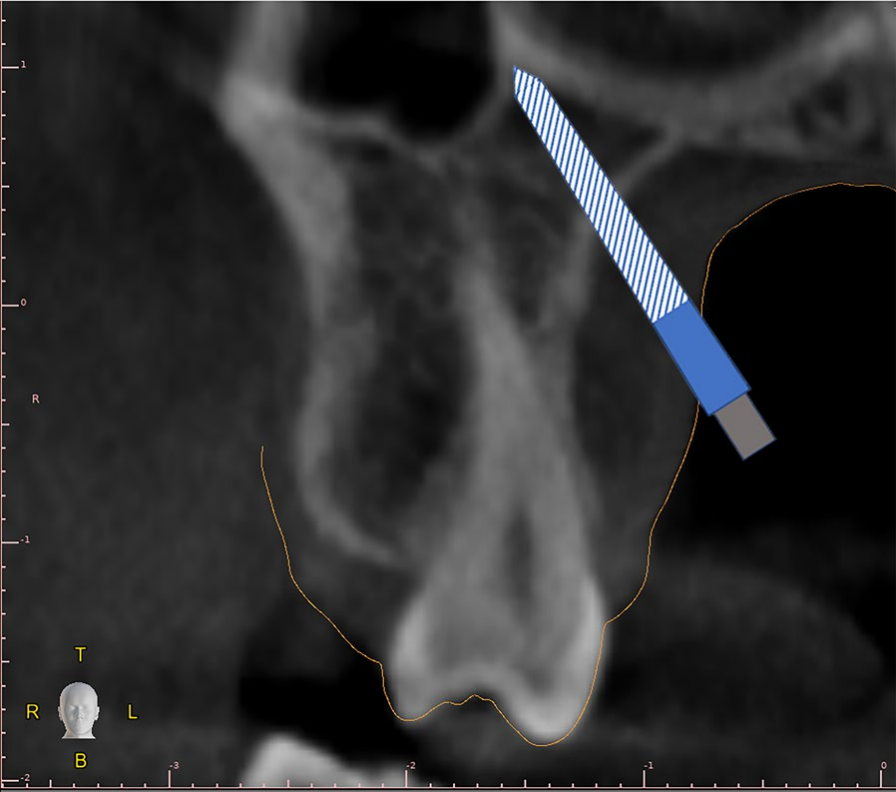
Ideal miniscrew position to reach tricorticalism stabilization. Light blue color shows ideal neck dimension extending to the transition zone between palatal mucosa and oral cavity. Gray color represents the miniscrew head interacting with the abutment of the palatal expander

The ideal miniscrew placement in the PPSAIS could be obtained when TADs placement is performed perforating cortical plate of the palatal vault (Fig. 4) and placing miniscrew tip in contact with nasal and maxillary sinus cortical plates [15, 16].

This approach could offer tricorticalism stabilization and the application of higher apical expansion force, thus improving biomechanics force application and potentially achieving better skeletal treatment effects (Fig. 4). On this regard, some authors proposed a miniscrew CBCT planning performed with the aim to reach the above-mentioned tricorticalism TADs placement approach [16].

Cortical bone characteristics are fundamentals for optimal miniscrew primary stability.

Results showed that the cortical bone of the palatine vault in different considered insertion sites may vary from 1.2 to 1.9 mm. These findings are coherent with previously published studies [5, 25, 26] and confirm an adequate amount of cortical bone for primary miniscrew stability.

Descriptive statistics showed cortical bone values tend to increase in the most caudal (+ 2P) and anterior (P2-M1) insertion sites (Tables 1 and 3). Moreover, data showed that insertion angle does not affect the amount of cortical bone thickness. Overall, quantitative differences of the cortical bone thickness of different evaluated insertion sites showed significant statistical differences (Table 1 and Additional file 1: Table S2). However, these differences could be clinically not significant.

The appraisal of palatal mucosa thickness usually presents difficulties in retrospective CBCT studies.

When lingual dorsum and palatal mucosa are in mutual contact, during CBCT examination, it is not possible to distinguish them from each other during subsequent imaging evaluation. Different methods have been proposed to overcome these limitations [27, 28]. In this study, we used a validated approach [29] matching the CBCT volume data and the digital models by means of suitable software. This methodology allowed to identify distinctly the profile of the palatal mucosa in all the analyzed cross-sectional scans.

The characteristics of the palatal mucosa are crucial for proper miniscrew selection, as the extent of mucosal thickness directly affects miniscrew length selection.

The findings of this study showed that at the level PPSAIS palatal mucosa is particularly thick with average values ranging from 4 to 7 mm (Tables 1 and 4). Palatal mucosa thickness showed no clinically significant differences (Table 1 and Additional file 1: Table S3) comparing different sagittal (P2-M1, M1F, M1-M2) and vertical insertion sites (− 2P, zeroP, + 2P). Data also showed that palatal mucosal thickness increases slightly with the inclination of the insertion axis relative to the occlusal plane (Tables 1 and 4).

This finding could be related to the geometric relationship of the insertion axes with the palatal mucosa layer rather than with a real anatomic increment of the mucosa thickness. However, this relative increment of mucosa thickness in high angle insertion axes has clinical relevance. Miniscrew inserted at a high angle to the palatal plane passing through thicker palatal mucosa should be longer and with the longest neck extension (Fig. 4). So, the proper selection of these miniscrew features is important to ensure adequate bone penetration and warrant optimal neck adaptation.

Finally, results showed that vertical skeletal growth pattern significantly affects considered outcomes. Hypodivergent subjects showed on average 1 mm of additional total bone depth, 0.2 mm of supplementary cortical bone, and 0.5 mm of reduced mucosa thickness compared to mesodivergent and hyperdivergent high angle patients. This data provides important information that can be relevant for optimal patient selection. Further studies could be necessary to better estimate the impact of skeletal characteristics on specific insertion sites of palatal posterior supra-alveolar bone.

## Conclusions

- The PPSAIS is an appropriate site for posterior insertion of palatal miniscrews.
- Total bone thickness seems to be optimal between the second premolar and the first molar with 45° angulation to the palatal plane; it seems to increase in most caudal insertion sites and in hypodivergent subjects.
- Cortical bone thickness is adequate for primary miniscrew stability.
- Mucosa thickness is on the average well represented; its extension ultimately affects miniscrew length selection.
- Considering high individual variation preliminary CBCT evaluation is important to achieve optimal miniscrew placement.

## Supplementary Information

**Additional file 1**. **Supplementary Table 1.** Inferential Statistics of Total Bone Depth (TBD). **Supplementary Table 2.** Inferential Statistics of Cortical Bone Thickness (CBT). **Supplementary Table 3.** Inferential Statistics of Mucosa Thickness (MT).

## Data Availability

The datasets used and analyzed during the current study are available from the corresponding author on reasonable request.

## Abbreviations

CBCT: Cone beam computer tomography
TADs: Temporary anchorage devices
PPSAIS: Palatal posterior supra-alveolar insertion site
PSS: Palatal slope segment
TBD: Total bone depth
MT: Mucosa thickness
CBT: Cortical bone thickness
ICC: Intraclass correlation coefficient
DICOM: Digital Imaging and COmmunications in Medicine
STL: STereoLithography
