# Supplementary material for: Evaluation of bone depth, cortical bone, and mucosa thickness of palatal posterior supra-alveolar insertion site for miniscrew placement
Source: Prog Orthod. 2022 Jun 6;23:18. doi: 10.1186/s40510-022-00412-9 (PMC9167746; doi:10.1186/s40510-022-00412-9)
Supplement: Supplementary file 1 — Additional file 1. Supplementary Table 1. Inferential Statistics of Total Bone Depth (TBD). Supplementary Table 2. Inferential Statistics of Cortical Bone Thickness (CBT). Supplementary Table 3. Inferential Statistics of Mucosa Thickness (MT). [file 40510_2022_412_MOESM1_ESM.docx]

|  | | | | | | | | | | | | |  |  |
| --- | --- | --- | --- | --- | --- | --- | --- | --- | --- | --- | --- | --- | --- | --- |
|  |  |  |  |  |  |  |  |  |  |  |  |  |  |  |
|  |  |  |  |  |  |  |  |  |  |  |  |  |  |  |
|  |  |  |  |  |  |  |  |  |  |  |  |  |  |  |
|  |  |  |  |  |  |  |  |  |  |  |  |  |  |  |
| Supplementary table 1. Inferential Statistics of Total Bone Depth (TBD). | | | | | | | | | | | | | |  |
|  |  | 45° | | |  | 60° | | |  | 75° | | | |  |
|  |  | P2-M1 | M1F | M1-M2 |  | P2-M1 | M1F | M1-M2 |  | P2-M1 | M1F | M1-M2 | |  |
| Multiple Comparison |  | NS | NS | NS |  | NS | NS | NS |  | P<0,05 | P<0,05 | P<0,05 | |  |
| +2P Vs zeroP |  | NS | NS | NS |  | NS | NS | NS |  | NS | NS | NS | |  |
| zeroP Vs -2P |  | NS | NS | NS |  | NS | NS | NS |  | NS | NS | NS | |  |
| +2P Vs -2P |  | NS | NS | NS |  | NS | NS | NS |  | P<0,05 | P<0,05 | P<0,05 | |  |
|  |  |  |  |  |  |  |  |  |  |  |  |  | |  |
|  |  | Total Bone Depth | | | | | | | | | | | |  |
|  |  | 45° | | |  | 60° | | |  | 75° | | | |  |
|  |  | +2P | zeroP | -2P |  | +2P | zeroP | -2P |  | +2P | zeroP | -2P | |  |
| Multiple Comparison |  | NS | NS | NS |  | NS | NS | NS |  | P<0,05 | P<0,05 | P<0,05 | |  |
| P2-M1 Vs M1F |  | P<0,05 | P<0,05 | P<0,05 |  | NS | P<0,05 | P<0,05 |  | NS | P<0,05 | P<0,05 | |  |
| M1F Vs M1-M2 |  | NS | NS | NS |  | NS | NS | NS |  | NS | NS | NS | |  |
| M1-M2 Vs P2-M1 |  | P<0,05 | NS | NS |  | NS | NS | P<0,05 |  | NS | NS | P<0,05 | |  |
|  |  |  |  |  |  |  |  |  |  |  |  |  | |  |
|  |  | Total Bone Depth | | | | | | | | | | | |  |
|  |  | P2-M1 | | |  | M1F | | |  | M1-M2 | | | |  |
|  |  | +2P | zeroP | -2P |  | +2P | zeroP | -2P |  | +2P | zeroP | -2P | |  |
| Multiple Comparison |  | NS | NS | NS |  | NS | NS | NS |  | NS | NS | NS | |  |
| 45° Vs 60° |  | NS | NS | NS |  | NS | NS | NS |  | NS | NS | NS | |  |
| 45° Vs 75° |  | NS | NS | NS |  | NS | NS | NS |  | NS | NS | NS | |  |
| 60° Vs 75° |  | NS | NS | NS |  | NS | NS | NS |  | NS | NS | NS | |  |
|  |  |  |  |  |  |  |  |  |  |  |  |  | |  |
|  |  |  |  |  |  |  |  |  |  |  |  |  | |  |
| Kruskal-Wallis multiple comparison test and Dunn-Bonferroni post-hoc test were used. Insertion sites at the level of second premolar and first molar (P2-M1), at the furcation of first molar (M1F) and at the first molar and second molar (M1-M2) levels were compared. Insertion axes passing trough 3 different vertical landmarks were compared: zero point (zeroP), 2mm cranial to zeroP (-2P), and 2mm caudal to zeroP (+2P). Insertion axes with three different angulation (45°, 60°, and 75°) were compared. | | | | | | | | | | | | | |  |
|  |  |  |  |  |  |  |  |  |  |  |  |  |  |  |
|  |  |  |  |  |  |  |  |  |  |  |  |  |  |  |
|  |  |  |  |  |  |  |  |  |  |  |  |  |  |  |
|  |  |  |  |  |  |  |  |  |  |  |  |  |  |  |

| Supplementary table 2. Inferential Statistics of Cortical Bone Thickness (CBT). | | | | | | | | | | | | |  |
| --- | --- | --- | --- | --- | --- | --- | --- | --- | --- | --- | --- | --- | --- |
|  |  | 45° | | |  | 60° | | |  | 75° | | |  |
|  |  | P2-M1 | M1F | M1-M2 |  | P2-M1 | M1F | M1-M2 |  | P2-M1 | M1F | M1-M2 |  |
| Multiple Comparison |  | P<0,05 | P<0,05 | P<0,05 |  | P<0,05 | P<0,05 | P<0,05 |  | P<0,05 | P<0,05 | NS |  |
| +2P Vs zeroP |  | NS | NS | P<0,05 |  | P<0,05 | NS | NS |  | NS | NS | NS |  |
| zeroP Vs -2P |  | NS | NS | NS |  | NS | NS | P<0,05 |  | P<0,05 | NS | NS |  |
| +2P Vs -2P |  | P<0,05 | P<0,05 | P<0,05 |  | P<0,05 | P<0,05 | P<0,05 |  | P<0,05 | P<0,05 | NS |  |
|  |  |  |  |  |  |  |  |  |  |  |  |  |  |
|  |  | Cortical Bone Thickness | | | | | | | | | | |  |
|  |  | 45° | | |  | 60° | | |  | 75° | | |  |
|  |  | +2P | zeroP | -2P |  | +2P | zeroP | -2P |  | +2P | zeroP | -2P |  |
| Multiple Comparison |  | P<0,05 | NS | NS |  | NS | P<0,05 | NS |  | NS | NS | P<0,05 |  |
| P2-M1 Vs M1F |  | NS | NS | NS |  | NS | P<0,05 | NS |  | NS | NS | P<0,05 |  |
| M1F Vs M1-M2 |  | NS | NS | NS |  | NS | NS | NS |  | NS | NS | NS |  |
| M1-M2 Vs P2-M1 |  | P<0,05 | NS | NS |  | NS | NS | NS |  | NS | NS | P<0,05 |  |
|  |  |  |  |  |  |  |  |  |  |  |  |  |  |
|  |  | Cortical Bone Thickness | | | | | | | | | | |  |
|  |  | P2-M1 | | |  | M1F | | |  | M1-M2 | | |  |
|  |  | +2P | zeroP | -2P |  | +2P | zeroP | -2P |  | +2P | zeroP | -2P |  |
| Multiple Comparison |  | P<0,05 | P<0,05 | NS |  | NS | NS | NS |  | NS | NS | NS |  |
| 45° Vs 60° |  | P<0,05 | NS | NS |  | NS | NS | NS |  | NS | NS | NS |  |
| 45° Vs 75° |  | P<0,05 | NS | NS |  | NS | NS | NS |  | NS | NS | NS |  |
| 60° Vs 75° |  | NS | NS | NS |  | NS | NS | NS |  | NS | NS | NS |  |
|  |  |  |  |  |  |  |  |  |  |  |  |  |  |
|  |  |  |  |  |  |  |  |  |  |  |  |  |  |
|  | | | | | | | | | | | | |  |
| Kruskal-Wallis multiple comparison test and Dunn-Bonferroni post-hoc tests were used. Insertion sites at the level of second premolar and first molar (P2-M1), at the furcation of first molar (M1F) and the first molar and second molar (M1-M2) levels were compared. Insertion axes passing through 3 different vertical landmarks were compared: zero point (zeroP), 2mm cranial to zeroP (-2P), and 2mm caudal to zeroP (+2P). Insertion axes with three different angulation (45°, 60°, and 75°) were compared. | | | | | | | | | | | | |  |
|  |  |  |  |  |  |  |  |  |  |  |  |  |  |
|  |  |  |  |  |  |  |  |  |  |  |  |  |  |
|  |  |  |  |  |  |  |  |  |  |  |  |  |  |

| Supplementary table 3. Inferential Statistics of Mucosa Thickness (MT). | | | | | | | | | | | | |  |
| --- | --- | --- | --- | --- | --- | --- | --- | --- | --- | --- | --- | --- | --- |
|  |  | 45° | | |  | 60° | | |  | 75° | | |  |
|  |  | P2-M1 | M1F | M1-M2 |  | P2-M1 | M1F | M1-M2 |  | P2-M1 | M1F | M1-M2 |  |
| Multiple Comparison |  | NS | P<0,05 | P<0,05 |  | NS | NS | P<0,05 |  | NS | P<0,05 | P<0,05 |  |
| +2P Vs zeroP |  | NS | NS | NS |  | NS | NS | P<0,05 |  | NS | P<0,05 | P<0,05 |  |
| zeroP Vs -2P |  | NS | P<0,05 | NS |  | NS | NS | NS |  | NS | NS | NS |  |
| +2P Vs -2P |  | NS | P<0,05 | P<0,05 |  | NS | NS | NS |  | NS | P<0,05 | P<0,05 |  |
|  |  |  |  |  |  |  |  |  |  |  |  |  |  |
|  |  | Mucosa Thickness | | | | | | | | | | |  |
|  |  | 45° | | |  | 60° | | |  | 75° | | |  |
|  |  | +2P | zeroP | -2P |  | +2P | zeroP | -2P |  | +2P | zeroP | -2P |  |
| Multiple Comparison |  | P<0,05 | NS | NS |  | NS | P<0,05 | NS |  | NS | NS | NS |  |
| P2-M1 Vs M1F |  | NS | NS | NS |  | NS | NS | NS |  | NS | NS | NS |  |
| M1F Vs M1-M2 |  | P<0,05 | NS | NS |  | NS | NS | NS |  | NS | NS | NS |  |
| M1-M2 Vs P2-M1 |  | P<0,05 | NS | NS |  | NS | P<0,05 | NS |  | NS | NS | NS |  |
|  |  |  |  |  |  |  |  |  |  |  |  |  |  |
|  |  | Mucosa Thickness | | | | | | | | | | |  |
|  |  | P2-M1 | | |  | M1F | | |  | M1-M2 | | |  |
|  |  | +2P | zeroP | -2P |  | +2P | zeroP | -2P |  | +2P | zeroP | -2P |  |
| Multiple Comparison |  | P<0,05 | P<0,05 | P<0,05 |  | NS | P<0,05 | P<0,05 |  | NS | P<0,05 | P<0,05 |  |
| 45° Vs 60° |  | NS | NS | NS |  | NS | P<0,05 | P<0,05 |  | NS | P<0,05 | P<0,05 |  |
| 45° Vs 75° |  | P<0,05 | P<0,05 | P<0,05 |  | NS | P<0,05 | P<0,05 |  | NS | P<0,05 | P<0,05 |  |
| 60° Vs 75° |  | P<0,05 | P<0,05 | P<0,05 |  | NS | NS | P<0,05 |  | NS | NS | P<0,05 |  |
|  |  |  |  |  |  |  |  |  |  |  |  |  |  |
|  |  |  |  |  |  |  |  |  |  |  |  |  |  |
|  | | | | | | | | | | | | |  |
| Kruskal-Wallis multiple comparison test and Dunn-Bonferroni post-hoc test were used. Insertion sites at the level of second premolar and first molar (P2-M1), at the furcation of first molar (M1F) and at the first molar and second molar (M1-M2) levels were compared. Insertion axes passing through 3 different vertical landmarks were compared: zero point (zeroP), 2mm cranial to zeroP (-2P), and 2mm caudal to zeroP (+2P). Insertion axes with three different angulations (45°, 60°, and 75°) were compared. | | | | | | | | | | | | |  |
|  |  |  |  |  |  |  |  |  |  |  |  |  |  |
|  |  |  |  |  |  |  |  |  |  |  |  |  |  |
|  |  |  |  |  |  |  |  |  |  |  |  |  |  |
